# Health Literacy in People with Diabetes: An Evolutionary Concept Analysis Using Rodgers' Approach

**DOI:** 10.17533/udea.iee.v43n2e02

**Published:** 2025-07-16

**Authors:** Erielton Gomes da Silva, Alex dos Santos Silva, Camila Ferreira do Monte, Suzana Oliveira Mangueira, Renan Alves Silva, Mailson Marques de Sousa, Marta Miriam Lopes Costa, Lidiane Lima de Andrade

**Affiliations:** 1 Nurse, M.Sc. Email: erieltong001@outlook.com. Corresponding Author. https://orcid.org/0000-0001-6010-8329 Universidade Federal da Paraíba Brazil erieltong001@outlook.com; 2 Nurse. Email: alex.silva2@academico.ufpb.br. https://orcid.org/0000-0002-6986-3635 Universidade Federal da Paraíba Brazil alex.silva2@academico.ufpb.br; 3 Nurse. Email: camilamonteferreira@gmail.com. https://orcid.org/0000-0002-5658-3458 Universidade Federal da Paraíba Brazil camilamonteferreira@gmail.com; 4 University teacher, Nurse, Ph.D. Email: suzana.mangueira@academico.ufpb.br. https://orcid.org/0000-0003-0931-8675 Universidade Federal da Paraíba Brazil suzana.mangueira@academico.ufpb.br; 5 University teacher, Nurse, Ph.D. Email: renan.a.silva@ufcg.edu.br. https://orcid.org/0000-0002-6354-2785 Universidade Federal de Campina Grande Brazil renan.a.silva@ufcg.edu.br; 6 University teacher, Nurse, Ph.D. Email: mailson.sousa@academico.ufpb.br. https://orcid.org/0000-0002-8099-4310 Universidade Federal da Paraíba Brazil mailson.sousa@academico.ufpb.br; 7 University teacher, Nurse, Ph.D. Email: marthamiryam@hotmail.com. https://orcid.org/0000-0002-2119-3935 Universidade Federal da Paraíba Brazil marthamiryam@hotmail.com; 8 University teacher, Nurse, Ph.D. Email: lidiane.andrade@academico.ufpb.br. https://orcid.org/0000-0003-1015-9237 Universidade Federal da Paraíba Brazil lidiane.andrade@academico.ufpb.br; 9 Federal University of Paraíba, Paraíba, Brazil. Universidade Federal da Paraíba Federal University of Paraíba Paraíba Brazil; 10 Federal University of Campina Grande, Paraíba, Brazil. Universidade Federal de Campina Grande Federal University of Campina Grande Paraíba Brazil

**Keywords:** concept formation, health literacy, diabetes mellitus., formación de concepto, alfabetización en salud, diabetes mellitus., formação de conceito, letramento em saúde, diabetes mellitus.

## Abstract

**Objective.:**

To analyze the evolution of the concept of health literacy (HL) in people with diabetes mellitus (DM) according to the Rodgers approach.

**Methods.:**

The Rodgers approach was used, based on a scoping review that followed the steps of the JBI. Only methodologically clear studies that Only studies (articles) that clearly addressed HA in DM were included. Primary sources from health databases (e.g. PubMed, Scopus, Web of Science, SciELO, LILACS and BDENF) and grey literature (e.g. theses, dissertations and guides) were analysed via university repositories, Google Scholar, the Brazilian Digital Library of Theses and Dissertations and the CAPES Journal Portal (a Brazilian research support agency). The protocol was registered on the Open Science Framework (DOI: https://doi.org/10.17605/OSF.IO/Y2T3R).

**Results.:**

A total of 197 studies were selected, identifying seven key attributes of health literacy (HA): understanding, application, seeking, communication, critical appraisal, and sociocultural competencies related to diabetes. A total of 197 studies were selected to identify seven key attributes of health literacy (HA), such as understanding, application, seeking, communication, critical appraisal, and sociocultural competencies related to diabetes. Background factors (e.g., educational level and age) and outcomes (e.g., self-care, adherence, and glycemic control) were also examined.

**Conclusion.:**

HA has evolved from basic literacy to digital and multidimensional competence, which presents challenges in vulnerable populations. HA is essential for the autonomy and effective management of diabetes mellitus (DM), highlighting the need for personalized educational interventions, primarily in primary care.

## Resumen

**Objetivo.:**

Analizar la evolución del concepto de alfabetización en salud (AS) en personas con diabetes mellitus (DM) según el enfoque de Rodgers.

**Métodos.:**

Se utilizó el enfoque de Rodgers, basado en una revisión de alcance que siguió los pasos del JBI. Solo se incluyeron estudios metodológicamente claros (artículos) que abordaran la AS en DM. Se analizaron fuentes primarias en bases de datos de salud (PubMed, Scopus, Web of Science, SciELO, LILACS, BDENF) y literatura gris (tesis, disertaciones, guías) a través de repositorios universitarios, Google Scholar, Biblioteca Digital de Tesis y Disertaciones de Brasil, y el Portal de Periódicos de la CAPES, (agencia brasileña de apoyo a la investigación). El protocolo fue registrado en Open Science Framework (DOI https://doi.org/10.17605/OSF.IO/Y2T3R).

**Resultados:**

. Se seleccionaron 197 estudios, identificándose siete atributos clave de AS, como comprensión, aplicación, búsqueda, comunicación, evaluación crítica y competencias socioculturales relacionadas con la diabetes. También se observaron antecedentes (nivel educativo, edad) y consecuencias (autocuidado, adherencia, control glucémico).

**Conclusión.:**

La AS ha evolucionado de alfabetización básica a competencia digital y multidimensional, presentando desafíos en poblaciones vulnerables. Es esencial para la autonomía y el manejo eficaz de la DM, destacando la necesidad de intervenciones educativas personalizadas, principalmente en atención primaria.

## Resumo

**Objetivo.:**

Analisar a evolução do conceito de letramento em saúde (LS) em pessoas com diabetes mellitus (DM) segundo o enfoque de Rodgers.

**Métodos.:**

Utilizou-se o enfoque de Rodgers, baseado em uma revisão de escopo que seguiu os passos do JBI. Foram incluídos apenas estudos metodologicamente claros (artigos) que abordassem o LS em DM. Foram analisadas fontes primárias em bases de dados de saúde (PubMed, Scopus, Web of Science, SciELO, LILACS, BDENF) e literatura cinzenta (teses, dissertações, guias) por meio de repositórios universitários, Google Scholar, Biblioteca Digital de Teses e Dissertações do Brasil e o Portal de Periódicos da CAPES (agência brasileira de apoio à pesquisa). O protocolo foi registrado no Open Science Framework (DOI https://doi.org/10.17605/OSF.IO/Y2T3R).

**Resultados.:**

Foram selecionados 197 estudos, identificando-se sete atributos-chave do LS, tais como compreensão, aplicação, busca, comunicação, avaliação crítica e competências socioculturais relacionadas à diabetes. Também foram observados antecedentes (nível educacional, idade) e consequências (autocuidado, adesão, controle glicêmico).

**Conclusão.:**

O LS evoluiu do letramento básico para a competência digital e multidimensional, apresentando desafios em populações vulneráveis. É essencial para a autonomia e manejo eficaz da DM, destacando a necessidade de intervenções educativas personalizadas, principalmente na atenção primária.

## Introduction

Diabetes Mellitus (DM) is a chronic disease resulting from ineffective insulin production or absorption, leading to elevated glycemic levels and systemic repercussions that can culminate in death.^1^ Considered a public health issue, it is estimated that over 62 million people live with DM in the Americas, a significant proportion of whom remain unaware of their condition. DM ranks as the sixth leading cause of mortality and is the primary cause of blindness among individuals aged 40 to 74 years.^2^ The effectiveness of treatment for people living with DM depends on various factors, including patient education, age, personal beliefs, treatment complexity, and the daily impact of insulin therapy.^3^ The term health literacy (HL) refers to the set of cognitive and social skills necessary to understand health information for self-care or the care of others. The concept of HL first emerged in the United States in the 1970s. HL levels significantly influence treatment adherence, lifestyle modifications, and adverse clinical outcomes related to DM.^4^^-^^7^

In Brazil, the term has evolved over time, also being referred to as health literacy, health literacia, or health education. ^7^ Regardless of terminology, HL is recognized by the World Health Organization (WHO) as a social determinant of health and has been widely promoted as a valuable tool for supporting health promotion strategies and patient-centered educational interventions.^8^^-^^10^ Concept analyses play a crucial role in refining and clarifying ambiguous concepts with multiple applications or limited definitions in the literature.^11^ While prior studies^12^^-^^16^ have analyzed the concept of HL and its relationship with DM,^17^^-^^19^ no research to date has focused explicitly on HL among people living with DM, particularly from Rodgers’ evolutionary perspective.

Health literacy has been widely applied in studies involving individuals with chronic conditions such as diabetes mellitus, cardiovascular diseases, and hypertension, especially in the contexts of self-care, disease management, and treatment adherence.^1^ Research indicates that the most frequently studied populations include older adults, individuals with lower educational levels, and those with low socioeconomic status-groups generally more vulnerable to the consequences of limited health Literacy.^2^^,^^3^ Adequate health literacy is associated with several facilitating factors, such as access to health services, higher education, social support, and effective communication with healthcare professionals. These factors contribute to improved self-care, treatment adherence, and health Outcomes.^4^

Despite the growing number of studies, there remains a lack of conceptual clarity and consistency in the definitions, attributes, and frameworks used to describe health literacy in people with diabetes. This fragmentation underscores a gap in the literature that justifies the need for a rigorous concept analysis.^1^^,^^4^ Additionally, as this is a scoping review, no quality assessment of the included studies was performed, in accordance with methodological recommendations prioritizing evidence mapping over critical appraisal.^5^^,^^6^

 The rationale for employing Rodgers’ evolutionary method lies in its ability to facilitate a comprehensive understanding of a concept.^20^ Rodgers’ method was chosen for this research because it focuses on how HL concepts evolve over time and across different settings, making it particularly suitable for examining the dynamic and multidimensional nature of health literacy in diabetes. Clarifying the concept of HL in this context is essential for a deeper understanding of the phenomenon, as well as for exploring its evolution across historical, political, and cultural dimensions. Given the influence of HL on health outcomes, a contemporary global understanding of this concept can provide theoretical foundations for its application in clinical practice. Such an analysis also differentiates HL in DM from other HL frameworks, allowing for the development of personalized interventions.

In line with the first and second steps of Rodgers’ evolutionary concept analysis, this study identifies health literacy in people living with diabetes mellitus as the central concept. The analysis is situated within the context of chronic disease management, with a specific focus on primary health care and patient education. This domain was selected due to the growing relevance of HL as a determinant of self-care capacity and treatment outcomes in chronic conditions such as DM. For nursing, this study provides guidance for professional practice aimed at health promotion, DM prevention, and the effective management of educational interventions through strengthened HL. This study aimed to analyze the evolution of the concept of HL among people living with DM using Rodgers’ evolutionary approach.

## Methods

The concept analysis guided by Rodgers’ evolutionary approach views concepts as continuous cycles that evolve over time.^20^ According to this model, six complementary and interdependent stages were followed: (1) identify the concept of interest; (2) select an appropriate domain for material selection; (3) analyze the extracted data to determine the attributes and contextual basis of the concept; (4) explore the data, distinguishing the characteristics of the concept, its antecedents, and consequences; (5) characterize substitute terms, related concepts, and a model case of the concept; and (6) formulate hypotheses and implications for the concept’s future development through discussion.

To investigate the concept of interest, a scoping review was conducted in accordance with the methodological recommendations of the JBI^21^ and reported following the PRISMA-ScR (Preferred Reporting Items for Systematic Reviews and Meta-Analyses extension for Scoping Reviews) checklist.^24^ Notably, a preliminary search on the Open Science Framework, Database of Abstracts of Reviews of Effects (DARE), and The Cochrane Library did not identify any scoping reviews on a similar topic. Consequently, the research protocol was registered on OSF. 

The mnemonic PCC was employed to guide the scoping review: P (Population) - people living with diabetes; C (Concept) - health literacy; and C (Context) - not restricted to a specific context to allow for a comprehensive analysis of the concept across various scenarios. Based on this framework, the guiding research question was formulated: “How has the concept of health literacy evolved in people with Diabetes Mellitus according to Rodgers’ evolutionary approach?”. 

Regarding eligibility criteria. Included were: (1) studies addressing the concept of health literacy in the context of people living with diabetes mellitus; (2) full-text availability in Portuguese, English, or Spanish; (3) publication between 1970 and 2024; and (4) any study design, including quantitative, qualitative, mixed-methods, and review articles, as well as theses, dissertations, and official guidelines. The exclusion criteria were: (1) studies that did not explicitly address the concept of health literacy in relation to diabetes mellitus; (2) duplicate records; and (3) letters to the editor, abstracts without full text, and opinion pieces without methodological clarity. 

Although the inclusion of studies in all languages is ideal to ensure maximum comprehensiveness, this review included only studies published in Portuguese, English, and Spanish. This decision was based on the language proficiency of the research team, which ensured accurate screening and interpretation of the selected materials. In line with JBI guidance, restricting the search to languages in which the reviewers are fluent is an accepted practice in scoping reviews, as it helps maintain methodological rigor and reliability of the analysis.^21^ Additionally, these three languages cover the majority of scientific output on the topic in the databases searched. The search focused on publications about health literacy from 1970 to 2024, reflecting the first documented use of the term.^22^

The databases used for the search were: Scopus, Web of Science (WOS), MEDLINE (via PubMed), Scientific Electronic Library Online (SciELO), Latin American and Caribbean Health Sciences Literature (LILACS), and BDENF, accessed through the Virtual Health Library (VHL) and the CAPES Periodicals Portal via Federated Academic Community (CAFe Access). For gray literature, repositories such as the Scientific Open Access Repositories of Portugal (RCAAP), the CAPES Periodicals Portal, the Brazilian Digital Library of Theses and Dissertations (BDTD), and Google Scholar were utilized, capturing the first 200 results.^23^ The search strategy was developed based on a preliminary exploration of the topic in two databases: MEDLINE (via PubMed) and LILACS. To ensure comprehensive searches, both controlled and uncontrolled descriptors were used following the PCC strategy. Controlled descriptors were extracted from the Health Sciences Descriptors/Medical Subject Headings (DeCS/MeSH), while uncontrolled descriptors included synonyms. These terms were combined using the Boolean operators OR and AND. Table 1 provides an overview of the initial search strategy, identified keywords, and the final search strategy implemented for each database. The search was carried out during the months of July to October 2024.

**Table 1:** Terms and search strategies used in the study.

MNEMONIC	DECS/MESH
P - People with Diabetes	Diabetes Mellitus; Diabete; Diabete Melito; Diabetes; Diabetes Melito
C - Health Literacy C- Health	Letramento em Saúde; Cultura em Saúde; Cultura sobre Saúde; Health Literacy; Alfabetización en Salud
	
LANGUAGE/DATABASE	INITIAL SEARCH STRATEGY
Portuguese (LILACS)	“Letramento em Saúde” OR “Cultura em SAúde” OR “Cultura sobre Saúde” AND “Diabetes Mellitus” OR Diabete OR “Diabete Melito” OR Diabetes OR “Diabetes Melito”
English (PubMed/LILACS)	*“Health Literacy” AND “Diabetes Mellitus”*
Spanish (LILACS)	*“Alfabetización en Salud” AND “Diabetes Mellitus”*
	IDENTIFIED TERMS FROM INITIAL SEARCH
-	Literacia em Saúde; Alfabetização em Saúde
DATABASE / LANGUAGE	FINAL SEARCH STRATEGY
PUBMED (English)	*("Health Literacy") AND ("Diabetes Mellitus")*
SCIELO (Portuguese, English, Spanish)	("Letramento em Saúde") OR ("Cultura em Saúde") OR ("Cultura sobre Saúde") OR ("Literacia em Saúde") OR ("Alfabetização em Saúde") AND ("Diabetes Mellitus") OR (Diabete) OR ("Diabete Melito") OR (Diabetes) OR ("Diabetes Melito")
	*("Health Literacy") AND ("Diabetes Mellitus")*
	*("Alfabetización en Salud") AND ("Diabetes Mellitus")*
WOS	*“Health Literacy” (All Fields) and “Diabetes Mellitus” (All Fields)*
SCOPUS	*“Health Literacy AND “Diabetes Mellitus”*
BDENF (via bvs) (Portuguese, English, Spanish)	*("Health Literacy") AND ("Diabetes Mellitus")*
	*("Alfabetización en Salud") AND ("Diabetes Mellitus")*
	("Letramento em Saúde") OR ("Cultura em Saúde") OR ("Cultura sobre Saúde") OR ("Literacia em Saúde") OR ("Alfabetização em Saúde") AND ("Diabetes Mellitus") OR (Diabete) OR ("Diabete Melito") OR (Diabetes) OR ("Diabetes Melito")
LILACS (via bvs) (Portuguese, English, Spanish)	*("Health Literacy") AND ("Diabetes Mellitus")*
	*("Alfabetización en Salud") AND ("Diabetes Mellitus")*
	("Letramento em Saúde") OR ("Cultura em Saúde") OR ("Cultura sobre Saúde") OR ("Literacia em Saúde") OR ("Alfabetização em Saúde") AND ("Diabetes Mellitus") OR (Diabete) OR ("Diabete Melito") OR (Diabetes) OR ("Diabetes Melito")
CAPES Journals	“Letramento em Saúde” OR “Literacia em Saúde” OR “Alfabetização em Saúde” AND “Diabetes Mellitus”
BDTD	“Letramento em Saúde AND “Diabetes Mellitus”
GOOGLE SCHOLAR	"Letramento em Saúde" OR "Literacia em Saúde" OR "Alfabetização em Saúde" AND "Diabetes Mellitus"
RCAAP	"Letramento em Saúde" AND "Diabetes Mellitus"

**Figure 1:**
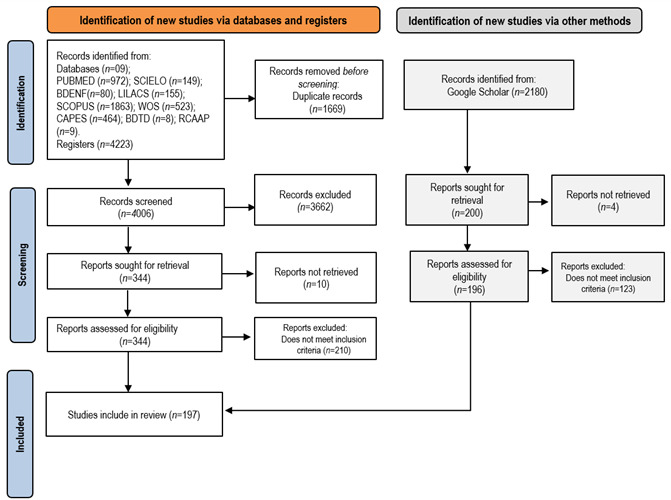
PRISMA Flowchart with the screening process for obtaining the sample

For the data analysis,^21^ references were imported into the Rayyan software, where they were analyzed in duplicate and independently. A third reviewer was consulted to resolve any conflicts. The study selection process was carried out in three stages. The first stage involved reading titles and abstracts, the second stage comprised a full-text review of eligible content for concept analysis, and the third stage involved retrieving the selected materials for subsequent data extraction. The entire process was illustrated using a flowchart following the PRISMA-ScR recommendations (Figure 1). It is important to note that no critical appraisal of the included studies was performed, as this step is not mandatory in scoping reviews. This approach is consistent with the methodological guidance provided by the JBI, which emphasizes mapping the evidence rather than assessing study quality.^21^

Data extraction was conducted using an instrument divided into two parts. Part 1 focused on characterizing the selected studies, including the study title, authors, type of material (article, dissertation, thesis, or other document), year of publication, study design (type of research), and setting and location of the study. Part 2 addressed specific questions for the concept analysis, namely: "What are the antecedents?", "What are the consequences?", "What attributes/concepts were identified?", "What substitute terms were identified?", and "What related concepts were identified?" The results were presented using tables and figures, and the data were analyzed descriptively, following Rodgers' framework,^20^ to meet the research objectives.

In addition to the conceptual categorization aligned with Rodgers’ model, a descriptive quantitative analysis was conducted to present the frequency and percentage of the attributes, antecedents, and consequences identified in the included studies. This allowed for the identification of the most recurrent elements of the concept. To illustrate the temporal evolution of the concept, a timeline was developed using extracted data about how the concept of HL in DM was addressed in each period. This tool helped synthesize conceptual changes and emerging dimensions between 1997 and 2024.

No critical appraisal of the included studies was performed, as this step is not required in scoping reviews. This decision aligns with the JBI methodological guidance, which emphasizes mapping existing evidence rather than evaluating study quality.^21^

## Results

This section will present the characteristics of the selected studies and the findings derived from Rodgers' evolutionary method. Stages 1 and 2 have already been detailed in the introduction, objectives, and methods sections. Stage 6 will be addressed in the discussion. 

### Sample characterization

The process of identifying materials from databases and gray literature, applying eligibility criteria, and obtaining the sample of studies included in this analysis can be visualized in the PRISMA-ScR flowchart,^24^ presented in Figure 1.

A total of 197 materials were included, of which 179 (90.86%) were articles, 11 (5.58%) were dissertations, four (2.03%) were conference proceedings, one (0.5%) was a thesis, one (0.5%) was a monograph, and one (0.5%) was a Ministry of Health guideline. Although 197 studies were included in the final sample, only 60 references are listed at the end of the article. This is because the analysis was carried out using a database developed by the research team, which compiled data extracted from all included studies. This approach allowed the authors to systematize the findings and present the synthesized results in tables and the discussion section. The references cited in the article correspond only to those directly used to support the theoretical framework, discussion, and other central parts of the manuscript. Therefore, not all included studies are cited individually, although all contributed to the analyzed dataset.

Regarding the methods used, 153 (77.66%) were cross-sectional studies, mostly descriptive and some analytical; 28 (14.21%) were literature reviews, including integrative, scoping, systematic, and narrative reviews; six (3.05%) were longitudinal studies, referring to the temporal design but primarily using observational or cohort methods; two (1.02%) were experimental studies, including one randomized clinical trial and one quasi-experimental study; one (0.5%) was a quantitative-qualitative study, one (0.5%) was a mixed methods study, one (0.5%) was an editorial, one (0.5%) was an opinion article, and one (0.5%) was a Ministry of Health guideline. The locations where the studies were conducted in Asia (36%) North América (26.2%), Europe (18%), South America (15.7%), Africa (2.3%) and Oceania (1.5%), being the three countries with the highest production: United States of America (21.32%), Brazil (12.69%) y Iran (7.11%).

**Figure 2:**
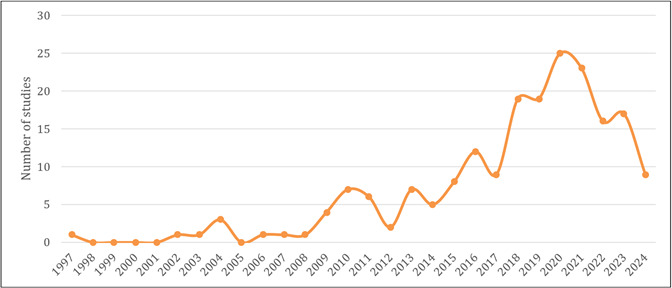
Distribution of the number of studies by year of publication

Regarding the years of publication, the first record of the concept of health literacy in people living with DM was observed in 1997 (*n=*01; 0.5%), and the highest number of materials found dates from the year 2020 (*n=*25; 12.7%). The distribution of the remaining study quantities by year of publication can be seen in Figure 2.

Regarding health environments, the locations where people were attended to were standardized by levels of health care, in studies where this information was available. It was observed that there was a greater interest in using the concept of HL in people living with DM in the context of secondary care, described in 60 (30.46%) of the identified studies. Next was primary care, with 22 (11.17%) studies, and finally, 23 (11.7%) works in the context of tertiary care.

### Attributes of the concept

From the collected data, seven attributes that form the concept of Health Literacy in people living with DM were identified. Among these, the most prevalent were "the ability to understand information about DM" (*n=*122; 61.9%) and "the ability to use knowledge to make appropriate health and DM-related decisions" (*n=*100; 50.7%). The other attributes can be seen in Table 2.

**Table 2:** Attributes of the health literacy concept related to people living with diabetes mellitus. (*n*=197)

Attributes	*N*	%
Ability to understand information about DM	122	61.9
Ability to use knowledge in making appropriate decisions about health and DM	100	50.7
Ability to seek information about DM from different sources (print and/or electronic)	62	31.5
Functional, cognitive, mental, and social competence in people living with DM	48	24.4
Communicative competence in people living with DM, including reading, writing, seeing, speaking, and hearing	39	19.8
Ability to critically evaluate information about health and DM	18	9.1
Having cultural knowledge in people living with DM	02	1.0

### Antecedents and consequents

After the data standardization process, it became possible to observe 28 antecedents for the concept under study and 29 consequents. The most prevalent antecedents were "higher education level" (32.9%), "younger age group" (26.4%), and "continuous educational interventions on DM" (17.26%). As for the most observed consequents, the following were noted: "better self-care related to DM" (56.85%), "adherence to treatments" (43.65%), and "better glycemic control" (32.9%). Others can be observed in Table 3. 

**Table 3:** Antecedents and consequents of the health literacy concept related to people living with diabetes mellitus (*n=*197)

Antecedents	%
Higher education level	32.9
Younger age group	26.4
Continuous educational interventions	17.26
Better socioeconomic and demographic conditions	15.23
Belonging to ethnic groups	8.63
Being female	7.61
Social/family support	7.11
Effective communication with professionals and people with DM	6.09
Having a job/employment	4.57
Good mental health	4.06
Fewer years since diagnosis	3.55
Access to Information and Communication Technologies	3.05
Good cognitive function	3.05
Self-efficacy	3.05
Knowledge about DM	2.54
Fluency in the language of the country of residence	2.54
Being married	2.54
Access to an environment sensitive to HL needs	2.03
Understanding the importance of self-management of DM	1.02
Qualified health professionals	1.02
Empowerment	1.02
Educational materials with clarity and organization, using short sentences and alternative titles divide the text	0.5
Fatigue	0.5
Access to healthcare	0.5
Perceived understanding to cope with the disease	0.5
Positive health beliefs	0.5
Having health insurance	0.5
Knowledge of HL competencies	0.5
Consequents	
Better self-care related to DM	56.85
Adherence to treatments	43.65
Better glycemic control	32.9
Utilization of healthcare services	23.86
Reduced DM complications	17.77
Critical understanding of disease, treatment, and complication prevention information through printed, virtual, and/or oral materials	17.26
Adequate knowledge about DM	14.21
Better health outcomes related to DM	10.15
Better self-efficacy	9.64
Effective communication and interaction with healthcare professionals	9.64
Higher quality of life	9.14
Critical, adapted, and specific education for self-care about DM	6.6
Reduced healthcare costs	4.57
Improved health behaviors	4.57
Reduced hospitalization	3.55
Better self-perception of health status	3.05
Increased empowerment	2.03
Greater motivation to make health decisions	2.03
Applying mathematical skills related to DM needs	2.03
Improved memory	1.52
Health equity	1.02
Effective self-assessment	1.02
Reduced risk factors for complications	1.02
Care planning centered on the needs of people living with DM	1.02
Planned health behavior	1.02
DM rehabilitation therapy	0.5
Reduced mortality	0,5
Increased trust in professionals	0.5
Acceptance of the disease	0.5

### Substitute terms, related concepts, and model case of the concept

**Figure 3:**
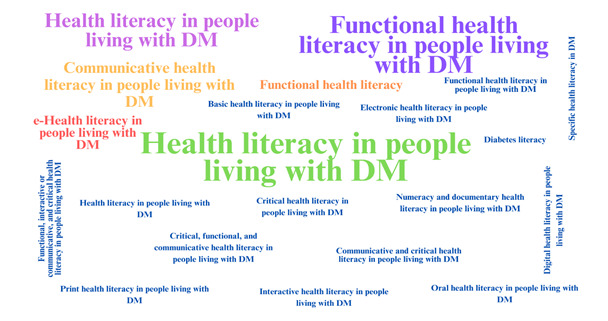
Substitute terms for the concept of health literacy related to people living with diabetes mellitus as identified in the literature (*n*=197)

Among the terms observed in the materials, the most prevalent was "Health Literacy" (*n=*64; 32.5%), followed by "Functional Health Literacy" (*n=*26; 13.20%), "Health Education" (*n=*12; 6.09%), and "Communicative Health Literacy" (*n=*3; 1.5%). These terms, along with the others presented in Figure 3, emerge for people living with DM as synonyms of the central term "Health Literacy," as their attributes interconnect and complement each other. This reflects the way the concept has evolved over time, despite its specificities.

### Related concepts

Clearly, the materials present four concepts related to HL in people living with DM, These are:(i) *Health numeracy (10.66%)*: A component of health literacy, essential for acting on health care based on numerical instructions. It involves the ability to understand and use numbers in everyday situations such as calculating medication dosage intervals, interpreting medication and food labels, determining insulin proportions, deciphering graphs, and weighing the risks of actions in health decision-making; (ii) *Self-efficacy (2.03%):* The people ability to trust and be motivated to make decisions about their health-related behaviors*;* (iii) *Self-care (0.5%) :* Problem-solving skills and disease management abilities, playing a crucial role in the successful management of diabetes*; and* (iv) *Empowerment (0.5%):* A feeling of power, control, and self-esteem, which fosters the interest and desire to participate in decisions about one’s own health.

### Model case of the concept

As proposed by Rodgers,^20^ a fictitious model case was created to illustrate the application of this concept in a possible practical reality: *Maria, a 55-year-old woman with low education, has been living with type 2 diabetes for a decade. She attends a group at the primary health care unit focused on health education about diabetes and hypertension. With the support of the nurse at this service, Maria acquired the essential HL skills related to her health condition. These include accessing, interpreting, and applying information obtained through printed and digital materials such as apps, games, and videos. As a result, this brought improvements to her chronic condition, helping her gain autonomy for self-care. Now, Maria can monitor her blood glucose levels, understands the importance of a balanced diet, physical activity, and adherence to prescribed medications as ways to maintain her health and prevent complications. Over time, she became an informal educator in the community, sharing her knowledge and encouraging family members and other participants in the primary care group to adopt healthy habits for improved quality of life and well-being*.

### Evolution of the health literacy concept in people living with diabetes based on Rodgers' approach

To illustrate how the concept of HL in people living with DM has evolved, a timeline was created. Firstly, it shows how the concept was addressed in the first three years of recorded data, as well as the emergence of the main related concept, numeracy. Next, the concepts are presented after the first and second decades since their emergence, along with the incorporation of digital/electronic media into this context. Given the larger number of publications in 2020 the need for data standardization was recognized to observe how the concept was addressed in that year. At the end of the timeline, a final synthesis is presented, reflecting the attributes acquired over time, up to the year 2024: 

1997 - First appearance of the concept. Reading and understanding health materials related to DM.

2002 - Second appearance of the concept. Reading and understanding, and acting according to medical instructions.

2003 - Third appearance of the concept and emergence of numeracy as a related concept. Remembering, reading, understanding, and adhering to information about DM. *Numeracy: Acting in health care based on numerical instructions. The ability to understand and use numbers in everyday situations related to DM and assess the risks of actions when making health decisions.*

2007 - Ten years after the first appearance of the concept. The ability to obtain, process, and understand basic health information and make appropriate health decisions. 

2017- Standardized concept after twenty years and emergence of electronic/digital competencies. The ability of individuals to obtain, read, understand, and evaluate information from various sources such as printed materials and/or the internet about diabetes to apply to their self-care.

2020 - Concept in the year with the most publications. The cognitive and social degree or capacity that determines the motivation individuals have to obtain, process, understand, communicate, evaluate, and apply health-related information to make assertive decisions about DM, reflecting on their self-care and the pursuit of prevention and treatment services.

2024 - Synthesis of the evolution of the concept until the current year. This is a determinant of health status and behaviors that goes beyond the competencies of common literacy. It is a set communicative, functional, cognitive, and social skills in people living with diabetes. These skills include numeracy, reading, writing, speaking, and listening, as necessary tools to motivate access to, understanding oof, critical evaluation, and utilization of information about the chronic condition. This is achieved through interpersonal contact, printed of electronic media in different contexts, with the purpose of improving self-care, resilience, individual and collective well-being, and disease management.

## Discussion

This study aimed to analyze the evolution of the concept of health literacy in people with diabetes using Rodgers’ evolutionary approach.^20^ The main findings identified seven core attributes, key antecedents such as higher education and younger age, and consequences like improved self-care, treatment adherence, and glycemic control. The analysis showed that the concept has evolved over time, expanding from basic literacy to a multidimensional construct that includes digital, communicative, cognitive, and social competencies essential for managing diabetes effectively.

The identification of better glycemic control, improved self-care, and greater treatment adherence as key consequences of HL highlights its critical role in DM management. These outcomes are essential not only for preventing acute and chronic complications-such as cardiovascular events, kidney failure, and diabetic foot-but also for reducing hospital admissions and healthcare costs. Moreover, individuals with adequate HL are more likely to engage in proactive health behaviors, follow clinical recommendations, and maintain a higher quality of life. Therefore, strengthening HL can significantly contribute to more sustainable and effective chronic disease care.

This includes information processing, decision-making, and the use of digital and numerical skills to practice self-care effectively. The relevance of applying this concept to clinical practice is evident through its own attributes. According to the attributes identified in this study, people who are capable of accessing, understanding, and evaluating information about DM, regardless of the form of access, to make informed decisions about their chronic condition assertively, can become autonomous in managing their self-care. In parallel, when this self-management is not feasible, people will have the communicative skills to address health issues and seek solutions.

Nevertheless, it is essential to consider that not all individuals have the capacity to access, understand, or evaluate health information autonomously.^38^ For these individuals, health literacy can still be developed through alternative pathways, such as relying on the support of healthcare professionals, family members, or community networks.^42^ In such contexts, HL becomes a shared or supported process, emphasizing the importance of relational and communicative aspects. This perspective reinforces the need for inclusive educational strategies that accommodate diverse literacy levels and promote equitable participation in self-care and decision-making.^34^^,^^39^^,^^40^^,^^48^

The identified antecedents highlight factors that can influence the acquisition of HL competencies in people living with DM. Among the most prevalent, it was observed that people with higher levels of education and younger populations tend to more easily incorporate new health information into their routines, including the use of digital technologies. In contrast, it is believed that these findings indicate that educational interventions focused on HL should prioritize groups with lower education levels and older populations. These people likely face greater barriers to accessing educational and technological resources.^38^^-^^40^ The identified consequences are directly linked to the antecedents of the concept. This inference can be observed as the exposure to educational programs dedicated to HL in DM leads to better disease management and a reduction in complications within this population. The findings further emphasize the need for clear, organized educational materials with short sentences, titles, and subtitles to divide the text, ensuring the efficiency of these programs. 

The data revealed that people living with DM who have a higher HL index are, primarily, better able to understand self-care, which facilitates adherence to treatment recommendations and ultimately leads to improved glycemic control.^34^^,^^41^ Another notable finding is the fact that this concept has been more explored in secondary care settings. This may indicate that patients receiving more specialized care are more exposed to educational interventions focused on HL for self-care in DM. Although the concept is relevant at all levels of care, it is important to emphasize the pivotal role of primary health care (PHC), which focuses on health promotion and disease prevention.^42^^,^^43^ Due to its proximity to the community, PHC has the potential to offer more effective tailored education for self-care, empowering people to better manage their health and prevent complications.^44^ For people living with DM, nurses in PHC play a key leadership role in managing the care provided.^45^ As health educators, they have the opportunity to promote HL in relation to DM by using intervention strategies that can take place through nursing consultations, group activities, home visits, and telephone follow-ups, for example.^46^ In doing so, they provide users with the tools necessary for effective disease management, treatment adherence, symptom recognition, and understanding clinical parameters.^9^^,^^47^

Regarding substitute terms, it is important to highlight that, although they may be seen as synonyms, they may have distinct focuses depending on cultural contexts, environments, the time period in which they were used, and/or the intended theoretical focus. For example, health literacy in people living with DM may refer to a broader set of skills for accessing, understanding, evaluating, and applying health information,^48^ while digital health literacy for the same population may be related to this skill set, but with an emphasis on virtual environments as the means of accessing information.^49^

As for the related concepts, self-efficacy and empowerment are fundamental factors that can support the application of HL skills in the daily lives of people living with DM. This is justified by the fact that, in addition to adequate HL, people with DM can gain the motivation needed to implement changes in their health behaviors when these factors are present.^50^^-^^51^ Similarly, health numeracy skills play an especially important role in supporting better control of the chronic condition.^52^ In the context of DM, numeracy functions as a component of HL and can influence peoples' ability to perform daily tasks, including insulin adjustments, proper glucose monitoring, and carbohydrate counting.^53^ Additionally, it helps them understand food labels, medications, and reduce hyperglycemic effects.^54^

The analysis revealed that the evolution of the HL concept for people with DM reflects an expansion of skills, beginning in 1997 with the simple reading and understanding of health materials, and progressing to a broader set of integrated competencies by 2024. The predominance of studies in 2020 may be associated with an increased awareness of HL in the context of self-care, largely due to the COVID-19 pandemic.^25^^,^^26^ This period brought new health concerns, amplified by the spread of misinformation, which highlighted the importance of HL, particularly for people with chronic conditions, such as DM.^27^

The concentration of studies in developed countries, which generally have greater access to healthcare and education,^28^^,^^29^ underscores the need to strengthen the assessment and research of health literacy in developing contexts. While education, information, and communication strategies may be implemented in many countries to improve self-care capacities among people with diabetes mellitus, the importance of measuring and studying these efforts has not been equally recognized or prioritized in all settings.^30^^,^^31^ In this regard, health education activities tailored to people living with DM remain crucial for achieving favorable health outcomes.^32^^,^^33^

A systematic review, which examined studies conducted over 24 years, concluded that people with DM who have good levels of health literacy (HL), or who participate in educational activities focused on developing HL for self-care, are able to maintain better blood glucose control and adopt more appropriate behaviors regarding their health condition.^34^ This reinforces the importance of health education, particularly with the nurse playing a participatory role in this process to promote a healthier and more balanced life for patients.^35^^,^^36^ For both theoretical understanding and practical application, it is important to recognize that this concept extends beyond traditional literacy. A scoping review that evaluated domains, levels, and contexts of HL from the perspective of the general population, much like this analysis, showed that HL is a complex and multidimensional concept, and it is essential to consider the nuances of each specific context.^37^

It is recommended that the psychosocial involvement (self-efficacy and empowerment) of people living with DM be assessed prior to the promotion of effective educational activities.^55^^,^^56^ This, combined with the use of materials tailored to people with inadequate HL, positively impacts glycemic control, medication adherence, effective DM self-care, and an improved quality of life.^57^^-^^60^

The findings contribute to the understanding of HL in the management of DM; however, certain limitations should be addressed in future studies. To nursing knowledge and practice by clarifying the attributes, antecedents, and consequences of HL in people living with DM. This understanding supports the development of more effective educational strategies and tailored interventions that nurses can implement in clinical settings, particularly in PHC. By promoting HL, nurses empower individuals to manage DM more autonomously, enhance treatment adherence, and reduce complications, ultimately improving care quality and health outcomes. The predominance of cross-sectional studies limits the understanding of causal relationships and the evolution of the concept. Longitudinal, experimental, and qualitative studies are recommended to assess changes, test interventions, and deepen patients' perceptions. The language restriction may have excluded relevant materials from regions such as Asia and Africa, where DM is prevalent, and approaches to HL may vary, although data collected from all continents provides a global perspective. 

Conclusion. This analysis shows that HL is vital for self-care and proper DM management. People with adequate HL have better glycemic control and adopt more effective management behaviors. The concept now includes digital skills for finding and evaluating information. Clarifying HL can guide health education tailored to people' HL levels, especially for those with limited access to health information. Integrating HL into health policies is key for self-care, preventing complications, and improving the quality of life for people with DM. Future research should include more languages, focus on primary health care for early interventions, and use interdisciplinary approaches with technology, psychology, education, and social support for culturally adapted and effective strategies.
